# Antimicrobial Activity of Cationic Antimicrobial Peptides against Gram-Positives: Current Progress Made in Understanding the Mode of Action and the Response of Bacteria

**DOI:** 10.3389/fcell.2016.00111

**Published:** 2016-10-14

**Authors:** Soraya Omardien, Stanley Brul, Sebastian A. J. Zaat

**Affiliations:** ^1^Department of Molecular Biology and Microbial Food Safety, Swammerdam Institute for Life Sciences, University of AmsterdamAmsterdam, Netherlands; ^2^Department of Medical Microbiology, Center for Infection and Immunity Amsterdam, Academic Medical Center, University of AmsterdamAmsterdam, Netherlands

**Keywords:** cationic antimicrobial peptides, mode of action, *Bacillus subtilis*, *Bacillus subtilis* spores, resistance mechanisms

## Abstract

Antimicrobial peptides (AMPs) have been proposed as a novel class of antimicrobials that could aid the fight against antibiotic resistant bacteria. The mode of action of AMPs as acting on the bacterial cytoplasmic membrane has often been presented as an enigma and there are doubts whether the membrane is the sole target of AMPs. Progress has been made in clarifying the possible targets of these peptides, which is reported in this review with as focus gram-positive vegetative cells and spores. Numerical estimates are discussed to evaluate the possibility that targets, other than the membrane, could play a role in susceptibility to AMPs. Concerns about possible resistance that bacteria might develop to AMPs are addressed. Proteomics, transcriptomics, and other molecular techniques are reviewed in the context of explaining the response of bacteria to the presence of AMPs and to predict what resistance strategies might be. Emergent mechanisms are cell envelope stress responses as well as enzymes able to degrade and/or specifically bind (and thus inactivate) AMPs. Further studies are needed to address the broadness of the AMP resistance and stress responses observed.

## Introduction

Most antibiotics used today are compounds that were discovered during the 1940s to 1960s (Lewis, 2013). With the rise of antibiotic resistance the search for alternative antibiotics became a priority to enable the treatment of imminent antibiotic resistant strains. It is in addressing this urgency that antimicrobial peptides (AMPs) have been proposed as a possible candidate for use as antimicrobial agents since their mode of action is presumed to be substantially different from existing antibiotics.

AMPs are, or are based on, natural molecules and are present in many organisms, ranging from microorganisms to humans, where they are an essential part of the innate immune system (Fox, 2013). The peptides have a broad-spectrum of activity as they are active against gram-positive and gram-negative bacteria as well as fungi (Wimley and Hristova, 2011). AMPs can be grouped based on their structure, which may be α-helical, β-sheet, cyclic, or adopt a more extended peptide conformation (Nguyen et al., 2011a,b; Wilmes et al., 2014). Extended peptides do not fold into a secondary structure (Nguyen et al., 2011b). Even though AMPs differ in sequence and structure, they share common features, which are their overall cationic charge, a significant fraction of hydrophobic residues and an ensuing amphipathic character (Nguyen et al., 2011b). It is the cationic properties that promote the preferential binding of AMPs to the negatively charged bacterial cytoplasmic membrane instead of the zwitterionic membrane of mammalian cells (Nguyen et al., 2011b). When the AMP reaches the lipid membrane interface of the target microorganism, the peptide takes an amphipathic conformation due to the hydrophobic residues (Papo and Shai, 2003; Bowdish et al., 2005; Teixeira et al., 2012), thus enabling the integration of the AMP into the membrane or the traversing thereof. AMPs usually disrupt the cytoplasmic membrane, but reports have been made of AMPs that seem to merely pass the membrane to target intracellular processes such as DNA, RNA, and protein synthesis (Park et al., 1998; Krijgsveld et al., 2000; Xiong et al., 2002).

Most research has been focused on the use of model membrane systems such as lipid vesicles, to determine the mode of action of AMPs. Even though this knowledge is essential in our understanding of the mode of action of AMPs, it does not fully explain their interaction with microbial membranes nor the response of microbes to the presence of AMPs. To address these two aspects, the current knowledge about the interaction of AMPs with bacterial cells and the response of bacteria to the presence of AMPs will be reviewed. Gram-positives are our main focus using *Bacillus subtilis* as model organism for pathogenic microbes such as *Staphylococcus aureus* and the spore forming *Clostridium difficile*. Knowledge concerning the antimicrobial activity of AMPs against gram-positive spores is limited and progress that has been made so far will be discussed. We briefly outline the cellular organization of gram-positive bacteria and spores of our model organism *Bacillus* to set the scene. Subsequently we will report on the cellular targets of AMPs and current knowledge about the response of gram-positives against AMPs. Information concerning gram-negatives will be presented wherever there is a lack of information about gram-positives bacteria.

## Gram-positive vegetative cell and spore composition

### Cell envelop of gram-positives

**Figure 1:**
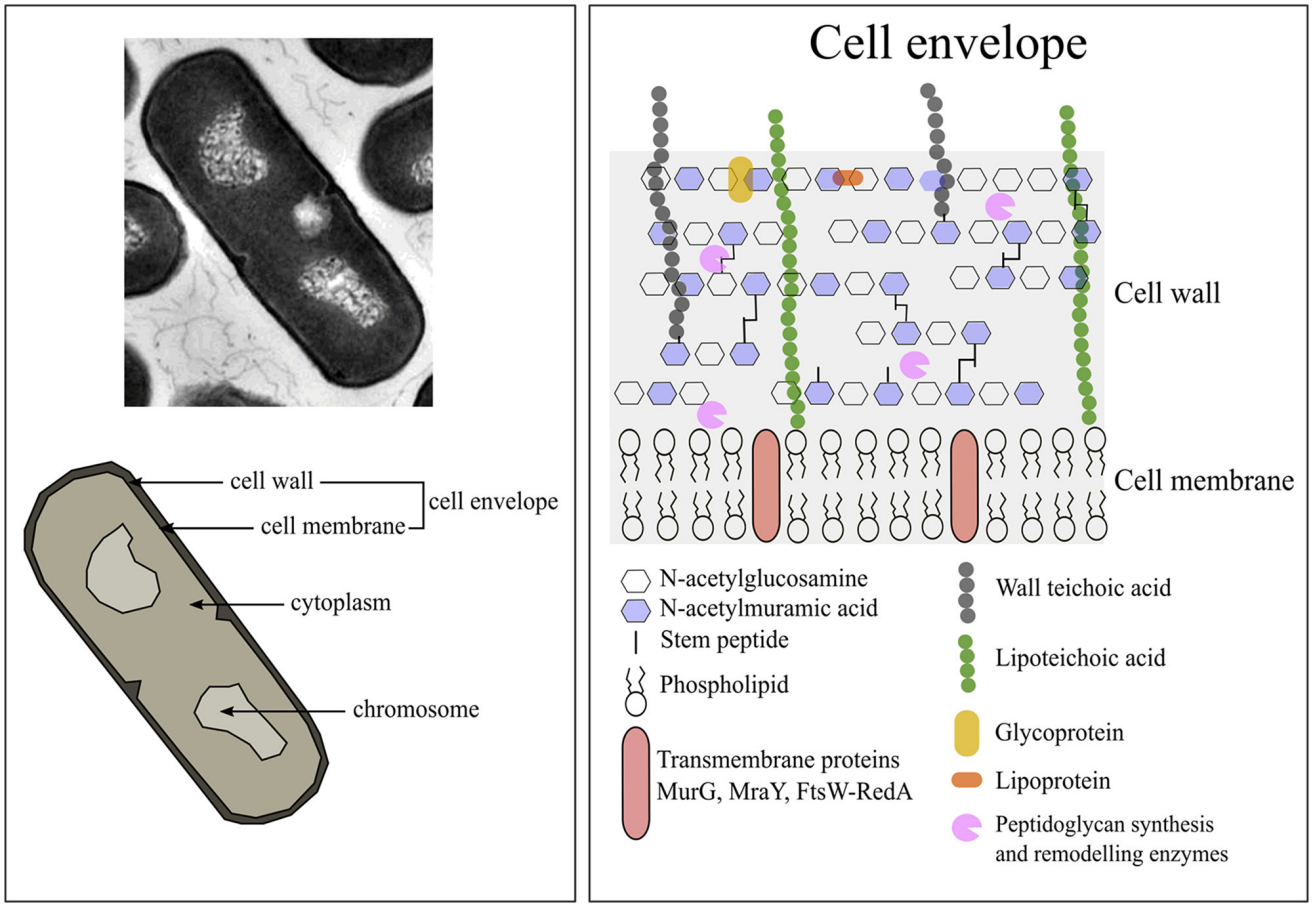
**The composition of ***Bacillus subtilis*** vegetative cells**. Image adjusted from Silhavy et al. (2010).

The cell envelope of a bacterium is the major line of defense against environmental threats. For gram-positives, the envelope consist of the cell wall and cytoplasmic membrane (Figure 1).

#### Cell wall of gram-positive bacteria

Compared to gram-negative bacteria, gram-positive species have a thicker cell wall of 30–100 nm width (Silhavy et al., 2010). The cell wall of *B. subtilis* consist of a thick peptidoglycan layer (±46% per dry cell weight) in which teichoic acids (±54% per dry cell weight) are embedded (Graham and Beveridge, 1994, and references therein). The protein fraction of the wall is ±10% of all cellular protein (Merchante et al., 1995). The *B. subtilis* cell wall structure is dynamic as it is continuously being synthesized and hydrolyzed during cell growth and cell division at the cytoplasmic membrane (Mobley et al., 1984; Merad et al., 1989; Graham and Beveridge, 1994; Banzhaf et al., 2012; Gray et al., 2015).

The peptidoglycan layer consists of linear glycan strands of alternating disaccharide-peptide repeats coupled through glycosidic bonds (Silhavy et al., 2010). These disaccharide-peptide repeats are of N-acetylglucosamine (GlcNAc) and N-acetylmuramic acid (MurNAc) residues and are coupled through β-1,4 glycosidic bonds (Vollmer et al., 2008). The glycan strands vary only slightly between different bacterial species, but the peptidoglycan differs considerably in the stem peptides composition and cross-links (Scheffers and Pinho, 2005). More information concerning the structure and synthesis of the cell wall can be found in various reviews (Scheffers and Pinho, 2005; Lee and Huang, 2013).

The teichoic acids comprise of wall teichoic acids and lipoteichoic acids (LTA). Wall teichoic acids are covalently connected to the peptidoglycan layer. LTA are macroamphiphiles that anchor in the membrane with their glycolipid and attach to the cell wall with their polyglycerol chains (Neuhaus and Baddiley, 2003). In *B. subtilis* teichuronic acid can also be found, but it is thought to only be present under low-phosphate conditions (Bhavsar and Brown, 2006).

#### Cell membrane of gram-positive bacteria

The cell membrane of *B. subtilis* in its stationary growth phase consists of protein (±62% per dry cell weight), membrane associated RNA (±22% per dry cell weight), and phospholipid (±16% per dry cell weight) (Bishop et al., 1967). According to the database *Subti*wiki 2.0 (http://subtiwiki.uni-goettingen.de/) about 721 proteins are localized at the cell membrane and have various functions. The phospholipid composition of the membrane of *B. subtilis*, of cells in their exponential growth phase in LB medium, consist of ±10% cardiolipin (CL), ±25% phophatidylglycerol (PG), ±50% phosphatidylethanolamine (PE), and ±15% lysyl-phosphatidylglycerol (lysyl-PG) (López et al., 2006). Cardiolipin and PG are negatively charged (−2 and −1, respectively), lysyl-PG positively charged (+1) and the other phospholipids are zwitterionic (neutrally charged) (Salzberg and Helmann, 2008). About 35% of the total membrane phospholipids contribute to the negative charge of the membrane, and only 15% contribute to the positive charge, therefore the net charge of the cell membrane is negative since the rest is zwitterionic (López et al., 2006).

Gram-positive bacteria are capable of modifying their membrane composition to increase or reduce the net charge. For instance, the membrane composition can change between the growth phase or between cultures grown in different media. *B. subtilis* cultured to its stationary growth phase has a membrane consisting of ±25% CL, ±40% PG, ±20% PE, ±15% lysyl-PG (López et al., 2006). Therefore, the negatively charged phospholipids increase by ±30% compared to the cell membrane compositions observed during the exponential growing phase. When cultured in LB medium with or without 1.5 M NaCl (high salinity), *B. subtilis* increased the relative amounts of cell membrane CL by ±20% decreasing the PE to ±17% (López et al., 2006). The PG content was similar (±24%) and lysyl-PG decreased only by ±5% (López et al., 2006). Therefore, the net negative charge of the cell membrane was increased.

### Cytoplasm of gram-positive bacteria

The cytoplasm of gram-positive bacteria mainly consist of the nucleoid and ribosomes. The nucleoid forms a dense central mass of DNA loops with the transcriptional machinery and ribosomes nearby (Lewis et al., 2000). The content of intracellular ribosomes, protein, RNA, or mobile genetic elements has, quantitatively, not generically been reported. Reported chromosome sizes of *B. subtilis* are 4215 kb (Logan and De Vos, 2015), of *S. aureus* 2814 kb (Kuroda et al., 2001), and of *C. difficile* 4290 kb (Sebaihia et al., 2006).

### Spores of gram-positive bacteria

Mainly bacteria from the genera *Bacillus* and *Clostridium* undergo the process of sporulation in response to nutrition limitation (Higgins and Dworkin, 2012). During sporulation some cells divide asymmetrically into a forespore and mother cell (Piggot and Hilbert, 2004). The result of sporulation is the production of a metabolically inactive or dormant endospore that is upon its release from the mother cell capable of resisting various environmental conditions such as extreme temperatures, desiccation, and ionizing radiation (Higgins and Dworkin, 2012). For more information concerning the sporulation process various reviews can be consulted (Piggot and Hilbert, 2004; Higgins and Dworkin, 2012; McKenney et al., 2013; Tan and Ramamurthi, 2014).

**Figure 2:**
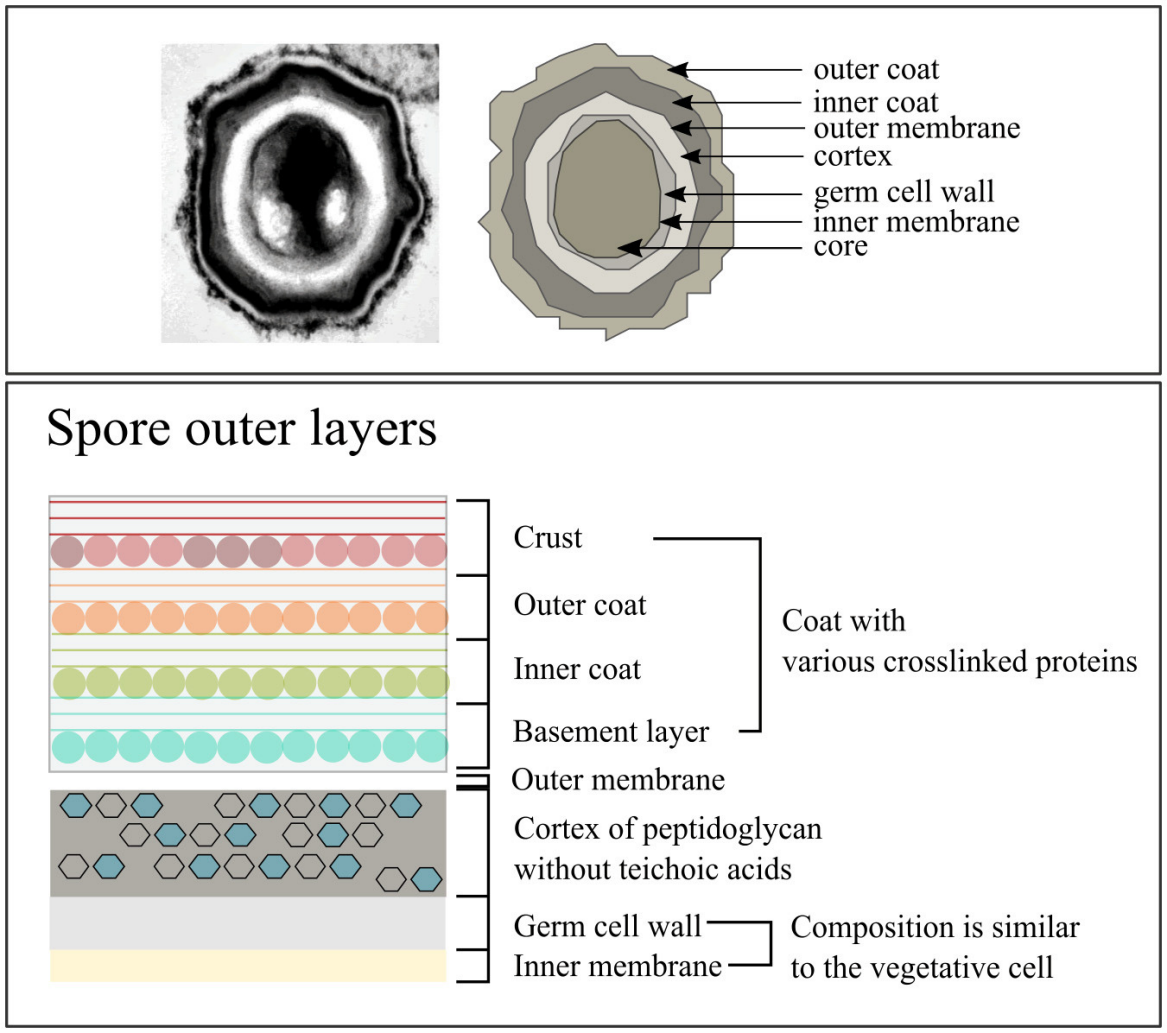
**The composition of ***Bacillus subtilis*** spores**. Image adjusted from McKenney et al. (2013).

The structure of spores differs from that of vegetative cells, consisting of exosporium (depending on the species), spore coat, outer membrane, a cortex, a germ cell wall (GCW), inner membrane, and central core (Figure 2) (Setlow, 2006). The exosporium consists mostly of proteins and is not found in all species, for instance it is absent in *B. subtilis* but present in *Bacillus cereus* and *Bacillus anthracis* (Setlow, 2006). Under the exosporium is the spore coat and in the case of *B. subtilis* the spore coat consists of two layers known as the outer and inner spore coat, which mainly consist of proteins (±30% of total spore proteins) (Driks, 1999; Henriques and Moran, 2000, 2007; Abhyankar et al., 2014). The spore coat protects the inner spore parts against various chemical and physical stresses (Henriques and Moran, 2007), but also interacts with the environment to facilitate the determination of favorable environmental conditions for initiation of spore germination (Henriques and Moran, 2007). Below the spore coat is the outer membrane, which is essential for spore formation (Piggot and Hilbert, 2004) but is not an important permeability barrier like the inner membrane (Setlow, 2006).

The spore cortex consists of two layers with the layer close to the inner membrane being the GCW (Popham, 2002). The GCW has a PG composition similar to the cell wall of vegetative cells. However, the thick outer PG layer covering the GCW is the cortex, which has different structural modifications compared to the vegetative cell wall (Popham, 2002). Prominent differences are the absence of teichoic acids and no crosslinking of the glycan strands (Popham, 2002). During germination the cortex is quickly degraded (Atrih et al., 1996; Popham et al., 1996; Atrih et al., 1998; Meador-parton and Popham, 2000; Popham, 2002) and the GCW becomes part of the vegetative cell wall (Atrih et al., 1996; Meador-parton and Popham, 2000). Cortex degradation results in the rehydration of the spore core, followed by metabolic activity and loss of the spore's protection against the external environment (Popham, 2002; Setlow, 2014).

The inner membrane of the spore is a resilient permeable barrier and is key in protecting the DNA within the core against damage (Setlow, 2006). The inner membrane is compressed and can double in volume during the initial stages of germination (Setlow, 2006). The lipid composition of the inner membrane is the same as that of vegetative cells but it is immobile and becomes fluid only during spore germination (Setlow, 2006). Zheng et al. (2016) have recently published an extensive proteomics study characterizing the inner membrane proteome and found that the protein composition of the inner membrane and of the vegetative cell membrane differs significantly.

The core of the spore contains macromolecules similar to those in vegetative cells, including DNA, ribosomes, and tRNA (Setlow, 2006). In contrast to vegetative cells, spores contain pyridine-2,6-dicarboxylic acid (dipicolinic acid, DPA) and small, acid-soluble spore proteins (SASP) (Setlow, 2006). DPA is 5–15% of the dry weight of spores and SASPs cover 3–6% of the total spore protein (Setlow, 2006). Both SAPs and DPA play a key role in protecting the DNA (Setlow, 2006) whilst in the spore. During germination DPA is released (Setlow, 2003) and during the early stages of spore outgrowth SASPs are degraded and used as carbon source for outgrowth (Setlow, 2006; Sinai et al., 2015).

## AMPs targeting the gram-positive cell wall

The anionicity of the surface of *B. subtilis* is determined by the presence of peptidoglycan and teichoic acids (Neuhaus and Baddiley, 2003) and thus the first contact of cationic AMPs with the bacterium is through electrostatic interaction. The anionic nature of the *B. subtilis* cell wall is proposed to be due to the presence of carboxyl groups of the muramyl peptides of peptidoglycan and the carboxyl and phosphate groups of the teichoic acids (Sonnenfeld et al., 1985). These anionic groups are positioned toward the outside of the cell wall (Sonnenfeld et al., 1985). The role that peptidoglycan plays, other than attracting the cationic peptide, is unknown. However, the importance of (derivatives of) teichoic acids in the binding of cationic AMPs became evident when the deletion of the *dlt* operon of *S. aureus* caused a reduction in efficacy of various AMPs against the bacterium (Peschel et al., 1999). The *dlt* operon mediates the addition of D-alanine esters to teichoic acids (Neuhaus and Baddiley, 2003).

Cell wall biosynthesis are, however, inhibited due to the binding of AMPs to lipid II, a cell wall synthesis precursor molecule. During the biosynthesis of the cell wall, uridine diphosphate (UDP)-MurNAc-pentapeptide is produced in the cytoplasm (Scheffers and Pinho, 2005; Bhavsar and Brown, 2006; Schneider et al., 2010). UDP-MurNAC-pentapeptide is transferred to a membrane acceptor bactoprenol, resulting in the formation of lipid I, which is converted to lipid II by the addition of GlcNAc from UDP-GlcNAc to MurNAc. Lipid II, after the addition of an interpeptide bridge in the case of gram-positives, is translocated to the outer side of the membrane and incorporated into the peptidoglycan chain. Similar to nisin, plectasin showed to bind to lipid II thus preventing its incorporation into the peptidoglycan chain (Schneider et al., 2010). Plectasin differed, however, from nisin as its hydrophobic part was located at the membrane interface whereas nisin inserts deep within the membrane bilayer to cause delocalization of lipid II (Hsu et al., 2004; Hasper et al., 2006; Schneider et al., 2010). Plectasin did not cause membrane damage or dissipate the membrane potential (Schneider et al., 2010). Human β-defensin 3 (hBD3) also showed to interfere with cell wall biosynthesis, without causing membrane damage. It prompted the accumulation of UDP-MurNAc-pentapeptide in the cytoplasm, triggered the formation of protrusions filled with cytoplasm through cell wall lesions, inhibited proteins involved in the formation of lipid II (FemX and penicillin-binding protein 2 [PBP2]), and bound to lipid II though electrostatic interaction (Sass et al., 2010). Similar cell wall biosynthesis inhibition due to lipid II binding was observed for α-defensin human neutrophil peptide-1 (HNP1) (De Leeuw et al., 2010). More information concerning the origin, characteristic, and target of peptides mentioned in the text can be found in Table 1.

**Table 1:** **Additional information concerning antimicrobial peptides and other antimicrobials mentioned in the texts**.

**Peptide**	**Origin**	**Characteristic**	**Target**	**Reference and references therein**
LL-37	Human	α-helical	Membrane	Sochacki et al., 2011
tPMP	Rabbit blood platelets	α-helical	Membrane	Xiong et al., 2005, 2006
Thrombocidin	Human blood platelets	α-helical,	Membrane	Krijgsveld et al., 2000
Defensin	Mammalian	α-helical, β-sheets	Membrane	Peschel et al., 2001
β-defensin 3 (hBD3)	Human	β-sheets	Cell wall	Sass et al., 2010
α-defensin neutrophil peptide-1 (HNP1)	Human	α-helical	Cell wall	De Leeuw et al., 2010
θ-Defensins	Old world monkeys leukocytes	Cyclic	Membrane	Wilmes et al., 2014
Protegrin (PG-1)	Porcine leukocytes	β-sheet	Membrane	Bellm et al., 2000; Bolintineanu et al., 2010
Cecropin A	*Hyalophora cecropia* (a moth)	α-helical	Membrane	Rangarajan et al., 2013
Daptomycin	*Streptomyces roseosporus*	Cyclic lipopeptide	Membrane	Steenbergen et al., 2005
Indolicin	*Bovine neutraphils*	Extended wedge-like conformation	Membrane	Staubitz et al., 2001; Nicolas, 2009
MP196	Synthetic hexapeptide	Linear peptide	Membrane	Wenzel et al., 2014, 2015
SMAP-29	Synthetic peptide derived from cathelicidin	α-helical	Membrane	Skerlavaj et al., 1999
Nisin	*Lactococcus lactis*	Lantibiotic	Membrane, cell wall biosynthesis	Bierbaum and Sahl, 2009
Subtilin	*Bacillus subtilis*	Lantibiotic	Membrane, cell wall biosynthesis	Bierbaum and Sahl, 2009
Gallidermin	*Staphylococcus gallinarum*	Lantibiotic	Membrane, cell wall biosynthesis	Kellner et al., 1988; Bonelli et al., 2006
Plectasin	*Pseudoplectania nigrella*	α-helical, β-sheet	Cell wall biosynthesis	Mygind et al., 2005; Schneider et al., 2010
Mersacidin	*Bacillus* sp. strain HIL Y-8,554,728	Lantibiotic	Cell wall biosynthesis	Brötz et al., 1995, 1998
Actagardine or Gardimycin	*Actinoplaes garbadinensis*	Lantibiotic	Cell wall biosynthesis	Brötz et al., 1995; Somma et al., 1977
Vancomycin	*Streptomyces toyocaensis*	Glycopeptide	Cell wall biosynthesis	Mascher et al., 2004
Bacitracin	*Bacillus* sps.	Cyclic dodecylpeptide	Cell wall biosynthesis	McDermott et al., 2003; Hiron et al., 2011
Mundticin KS	*Enterococcus mundtii*	Bacteriocin	Unknown (possibly membrane)	Kawamoto et al., 2002; Sakayori et al., 2003
SP1-1	Synthetic peptide	α-helical	Serine kinase inhibition	Dangel et al., 2013

AMPs also indirectly target the cell wall by triggering autolysis of bacterial cells, which is the process when cells release autolysins, that cleave peptidoglycan, resulting in the destruction of itself. LTA, located at the septum, regulate autolysins and have shown to release autolysins when disturbed by AMPs (Bierbaum and Sahl, 1985, 1987; Wilmes et al., 2014). The glycolipid anchor plays an important role in the inhibitory effect of LTA on autolysins (Fischer et al., 1981) and this is probably due to the disturbance of the cell membrane by AMPs causing a delocalization of the glycolipid anchor. The additional importance of anionic phosphodiester groups of LTA were established when replacing it with D-alanine caused the release of autolysins, but replacing the same positions with non-charged glycosyl residues had no effect on the inhibitory action of LTA. This finding is, however, in contrast with previous findings that reports that the addition of D-alanine to the teichoic acids reduces the efficacy of AMPs, as mentioned above. Lantibiotics, Pep5, and nisin, have shown to cause autolysis, and an AMP from Old world monkey leukocytes, θ-Defensins (Bierbaum and Sahl, 1985, 1987; Wilmes et al., 2014).

## AMPs' interaction with the gram-positive cell membrane

Cationic AMPs are attracted to the cell membrane through electrostatic interaction, therefore focus has been placed mainly on the phospholipid composition of cells. Various models have been proposed for the interaction of AMPs with membranes and have been extensively reviewed (Nguyen et al., 2011b; Wimley and Hristova, 2011). For example, the barrel-stave model suggest that peptides accumulate on the surface of the membrane and insert into the membrane when a threshold amount is reached (Nguyen et al., 2011b). The toroidal pore model proposes that a peptide-and-lipid-lined pore is formed whereas the disordered toroidal pore model suggests that the peptide causes pore formation stochastically requiring less peptides per inferred pore (Nguyen et al., 2011b). However, these models are based on studies performed on lipid vesicles and cannot fully explain the interaction of AMPs with the complex bacterial cytoplasmic membrane.

AMPs act on vegetative cells causing membrane damage and ensuing loss of transmembrane potential and essential molecules (Nakajima et al., 2003; Bolintineanu et al., 2010; Lee et al., 2015). Membrane damage has been shown to perturb cellular homeostasis leading to either increase in cellular volume (Bolintineanu et al., 2010) or cause shrinking of cells (Wang et al., 2013). Pore or channel formation in cells has been shown using fluorescent dyes such as propidium iodide (Wang et al., 2013; Garg et al., 2014) and Sytox® Green (Barns and Weisshaar, 2013). Abnormal septum formation (Friedrich et al., 2000; Nakajima et al., 2003) and blebbing (Skerlavaj et al., 1999) has been observed with TEM. Blebbing is the formation of membrane bulges when the cytoskeleton is detached from the cell membrane causing the latter to swell. In the case of *S. aureus* treated with SMAP-29, blebbing occurred frequently at the sites of cell division (Skerlavaj et al., 1999). Still, generally these studies only confirm membrane damage but do not indicate whether the membrane is the initial and only target of the AMP or if the peptide moves past the membrane into the cytoplasm to target other essential cellular function such as DNA and RNA synthesis.

Barns and Weisshaar (2013) attempted to explain the time resolved events in pore/channel formation by the membrane active peptide LL-37 in *B. subtilis* using single cell, live-imaging fluorescence microscopy. When a low concentration of LL-37 (2× MIC) was used the growth rate was decreased without causing membrane permeabilization, but a higher concentration of LL-37 (4 × MIC) caused cell shrinking and permeabilization. Some of the cells treated with 2 × MIC LL-37 had an altered growth rate but were able to recover. Cells treated with 4 × MIC LL-37 were permeabilized and unable to recover. These findings suggested that at a low concentration the peptide is able to interact with the membrane but causes reparable membrane damage. At a high concentration, a threshold is reached that causes irreversible membrane damage. The severe membrane damage that leads to cell shrinking or aberrant septum formation, probably occurs only at high peptide concentrations. Therefore, to determine the mode of action of peptides a concentration range should be selected from lethal to sublethal concentrations to obtain a broad mechanistic overview of how the peptide targets the cell.

In the above mentioned study, rhodamine dye labeled LL-37 (Rh-LL-37) did not have the same antimicrobial effects as the unlabeled LL-37 against *B. subtilis* and the mode of action could thus not be determined using microscopy. Rh-LL-37 was, however, active against *Escherichia coli* (Sochacki et al., 2011) presumably in a similar manner as the unlabeled peptide. The authors observed that Rh-LL-37 targeted the cells in three phases. Phase 1 was the fast binding of Rh-LL-37 to the outer membrane (OM), the lipopolysaccharides (LPS), and O-antigen layers. The translocation of the peptide over the OM required a threshold concentration of OM bound peptide. It also appeared that the peptide was able to move past the OM without causing severe local OM damage. *E. coli* growth stopped as soon as Rh-LL-37 entered the periplasmic region, which is known as phase 2. Rh-LL-37 entered the periplasm at the septal region, where it bound to immobile elements before it could move past the cytoplasmic membrane (CM). The immobile elements were suggested to be peptidoglycan. Phase 3 was the permeabilization of the CM, which occurred at the septum.

Rh-LL-37 preferably bound to the septating cells instead of the non-septating cells (Sochacki et al., 2011). Similar results were obtain for cecropin A (Rangarajan et al., 2013). In addition, cecropin A was also shown to target the new pole instead of the old pole (Rangarajan et al., 2013). It is known that the septum, new pole, and newly formed cells are rich in anionic phospholipids such as CL (Mileykovskaya and Dowhan, 2010). This CL-rich domain at the septum is known to recruit the DNA replication machinery and key cell division proteins such as FtsZ, FtsA, and ZipA (Lutkenhaus et al., 2012). It is conceivable that the binding of peptides to CL could cause dissociation of proteins localized at this region, thus having severe consequences on cellular homeostasis. A similar study still needs to be performed on *B. subtilis*, but it is conceivable that LL-37 or cecropin A also bind to the cell wall until a threshold is reached, pass the cell wall in an unknown manner to preferably bind to the membrane at the septum or new poles.

In another study using fluorescence microcopy, *B. subtilis* was exposed to sublethal concentrations of daptomycin which caused curvature in the structure of the membrane (Pogliano et al., 2012). Proteins that recognize negative membrane curvature such as the cell division protein DivIVA, are bound to these membrane sites. The combined effect of a curved membrane and DivIVA localized at these random curved membrane sites caused alterations in the cell wall and the formation of an additional septum (Pogliano et al., 2012). Distortion of the cell membrane and cell wall results in a weakening of these structures causing the membranes to extrude (forming blebs) (Pogliano et al., 2012). This could finally cause rupturing of the cell membrane and cell wall (Pogliano et al., 2012). Interestingly, *S. aureus* was shown previously to have an abnormal, asymmetric division septum, and additional septa in response to daptomycin (Cotroneo et al., 2008). This study performed by Pogliano et al. (2012) suggests that in addition to distorting the membrane, membrane bound proteins could be delocalized thus compromising the normal functioning of the cell.

A synthetic hexapeptide called MP196 was shown to cause the delocalization of membrane-bound proteins, such as MurG that is involved in lipid II biosynthesis (Wenzel et al., 2014). The delocalization of membrane-bound proteins was thought to be the result of an altered membrane potential. MP196 interact with the cell membrane without causing membrane damage or the efflux of ions, but accumulated intracellularly through an unknown manner. MP196 also targeted the respiratory chain by detaching cytochrome c from the bacterial membrane. Abrogation of respiratory chain activity subsequently reduced ATP synthesis and subsequently macromolecules biosynthesis (Wenzel et al., 2014).

Taken together these findings suggest that the local membrane concentration of the peptide plays a key role in the mode of action of AMPs observed in microscopy. Cardiolipin might play an important role in the interaction of the AMPs with the cell membrane that are present at the septum or new pole where AMPs accumulate. AMPs distort the membrane which leads to the delocalization of membrane localized proteins, such as MurG, contributing to the death of the cell. Thus, membrane-bound proteins might also play an important role in the mode of action of AMPs that has not been fully addressed.

## Other cellular targets of AMPs

It is conceivable that AMPs which disrupt the cellular membrane or translocate to the cytosol without causing membrane damage can bind to the abundant intracellular polyanionic molecules, such as the nucleic acids and proteins. For instance, thrombin-induced platelet microbicidal proteins (tPMPs), from rabbits, causes limited membrane permeabilization but inhibits DNA and RNA synthesis and hence indirectly protein synthesis (Yeaman et al., 1998; Xiong et al., 2002). Thrombocidin, from human blood platelets, was unable to dissipate the membrane potential thus also suggesting an intracellular target (Krijgsveld et al., 2000). Similar results were obtained for indolicidin and an AMP that causes limited membrane permeabilization and inhibits DNA replication as well as transcription by binding to the DNA duplex, preventing it from unwinding (Falla et al., 1996; Ghosh et al., 2014). A synthetic peptide (SP1-1), which was based on natural α-helical AMPs, was able to move past the cell envelope of *S. aureus* into the cytoplasm to interacts with the serine protein kinase RsbW, an anti-sigma factor (Miyazaki et al., 1999; Dangel et al., 2013). Various cell processes were affected such as the induction of cell wall metabolism, oxidative phosphorylation (cytochrome d), biofilm formation and virulence, and the repression of amino acid biosynthesis and ABC transporters involved in antibiotic resistance (Dangel et al., 2013).

## AMPs activity against gram-positive spores

To the best of our knowledge anti-spore activity has only been studied for subtilin produced by *B. subtilis* (Liu and Hansen, 1993) and for nisin from *Lactococcus lactis* (Gut et al., 2008). Nisin and subtilin were only active against germinated spores and the inner membrane appeared to be the main target (Liu and Hansen, 1993; Gut et al., 2008, 2011). Disruption of the inner membrane could be preventing the establishment of metabolism and the shedding of the spore coat (Gut et al., 2008), thus preventing outgrowth. In presence of either of the peptides, the germination receptors were activated, DPA released, water taken up and the cortex peptidoglycan hydrolyzed before the inner membrane was exposed to the AMPs. The importance of lipid II binding for the antimicrobial activity of nisin against spores was investigated. The results showed that outgrowth inhibition of *B. anthracis* spores by nisin was dependent on lipid II binding as with vegetative cells though in itself the association with the peptidoglycan precursor was not sufficient for inhibition to be effective (Gut et al., 2011).

## Theoretical interaction of AMPs with gram-positives (bionumbers and bioestimates)

Recently, quantification of cell(wall)-related compounds with respect to describing cellular physiology in interaction with its environment has been pursued more and more. Bionumbers and bioestimates have for instance been described with respect to the estimation of effects of culturing conditions on the physiology of *Saccharomyces cerevisiae* (Klis et al., 2014). Along the same vein, we explore here the theoretical amount of AMPs that can bind to or occupy a space on *B. subtilis* cells or spores. Thus, we aim to infer the cell components that AMPs could interact with. The length and width of *B. subtilis* vegetative cells were obtained from the Bionumbers database (http://bionumbers.hms.harvard.edu/) and that of the spores were obtained from results published by Leuschner and Lillford (2000). The area and volume of vegetative cells were determined by assuming it has a shape consisting of a cylinder and two half spheres. The spores were assumed to be ellipsoidal. The calculated area of vegetative cells was 12.9 μm^2^ in rich medium and 6.3 μm^2^ in minimal medium (Table 2). The volume of vegetative cells was 2.6 μm^3^ in rich medium and 1.6 μm^3^ in minimal medium. The area and volume of spores is 2.4 μm^2^ and 0.9 μm^3^. Calculations can be performed using the Planetcalc (http://planetcalc.com/149/).

**Table 2:** **Bioestimates of the interaction of LL-37 with ***Bacillus subtilis*** vegetative cells and spores**.

	***Bacillus subtilis*** **vegetative cell**		***Bacillus subtilis*** **spore**	
	**Formula used**	**Estimates**	**Formula used**	**Estimates**
Length (l)		4.7 μm or 2.3 μm^#^		1.4 μm^$^
Width (w)		0.87 μm^#^		0.55 μm^$^
Radius (r)	1/2w	0.44 μm	1/2w	0.28 μm
Volume^!^ (V_B.subtilis_)	V_sphere_ + V_cylinder_ = 4/3πr^3^ + πr^2^(l – w)	2.6 μm^3^ or 1.1 μm^3^	V_ellipsoid_ = 4/3πlwr	0.9 μm^3^
Area^!^ (A_B.subtilis_)	A_sphere_ + A_cylinder_ = 4πr^2^ + 2πr(l – w)	12.9 μm^3^ or 6.3 μm^2^	A_ellipsoid_ = 4π((lw)^1.6^+ (lr)^1.6^+ (wr)^1.6^)/3)1/^1.6^	2.4 μm^2^
Minimal inhibitory concentration (MIC)		1 μM^*^		1 μM^*^
Number of cells		1 × 10^6^^*^		1 × 10m^6^^*^
Volume		100 μl^*^		100 μl^*^
Avogadro's Number is		6.022 × 10^23^ per mol		6.022 × 10^23^ per mol
Amount of molecules required to inhibit (N)		6.0 × 10^7^ molecules per cell		6.0 × 10^7^ molecules per spore
Assumed size/radius of LL-37 (4 kDa)		0.001 μm		0.001 μm
Area of LL-37 (sphere)	A_LL-37sphere_ = 4πr^2^	1.5 × 10^−5^ μm^2^		1.5 × 10^−5^ μm^2^
Area of LL-37 (circle)	A_LL-37circle_ = πr^2^	3.1 × 10^−6^ μm^2^		3.1 × 10^−6^ μm^2^
Volume of LL-37 (sphere)	V_LL-37sphere_ = 4/3πr^3^	4.2 × 10^−9^ μm^3^		4.2 × 10^−9^ μm^3^
Amount of LL-37 that covers celll^ˆ^	A_hexagonal_ = 0.9(A_B. subtilis_/A_LL-37circle_)	3.7 × 10^6^ or 1.8 × 10^6^		
Amount of LL-37 that fills the cell^ˆ^	V_hexagonal = 0.6_(V_B. subtilis_/V_LL-37sphere_)	4.5 × 10^8^ or 1.9 × 10^8^		
Amount of LL-37 that covers the spore^ˆ^	A_hexagonal = 0.9_(A_B. subtilis_/A_LL-37circle_)			6.9 × 10^5^
Amount of LL-37 that fills the spore^ˆ^	V_hexagonal = 0.6_(V_B. subtilis_/V_LL-37sphere_)			1.7 × 10^8^

! *Assuming that the shape of Bacillus subtilis vegetative cell is a cylinder at the center and has two half spheres at the ends and Bacillus subtilis spores is an ellipsoidal shape*.

* *Minimal inhibitory concentration obtained from Barns and Weisshaar (2013) and the cell counts are an estimation of the OD_600_ 0.00025 cells used in the study*.

# *Lenght of B. subtilis is 4.7 μm in rich medium and 2.3 μm in minimal medium. Values obtained from http://bionumbers.hms.harvard.edu/default.aspx*.

$ *Values obtained from Leuschner and Lillford (2000)*.

ˆ *Assuming hexagonal close packing of circles or spheres. A value of 0.9 is used to compensate for hexagonal close packing of circles and a value of 0.74 is used for spheres*.

To estimate the number of AMPs that can bind to the surface and occupy the space of the cell or spore, membrane disrupting peptide LL-37 (4 kDa) was employed as an example. LL-37 displayed a MIC value of 1 μM against an inoculum of about 1 × 10^6^ cells (OD_600_ of 0.0025) in a 100 μl reaction (Barns and Weisshaar, 2013). The same was assumed for spores. The number of LL-37 molecules that can target each *B. subtilis* cell or spore was estimated to be about 6.0 × 10^7^ molecules. Based on the calculations of Erickson (2009) of a 5 kDa protein, we estimated that the 4 kDa LL-37 has a spherical shape with a radius of about 1 nm. This made it possible to calculate, for a rough order of magnitude estimation, the number of LL-37 that can bind to the surface of the vegetative cell or spore, or fill the intracellular compartments. An hexagonal close packing formation of the LL-37 molecule was assumed and the highest density of the arrangement of circles is 0.9 and of spheres 0.74 (Wells, 1991; Steinhaus, 1999; Chang and Wang, 2010). By dividing the area of the cell surface by the area of the LL-37 molecule, a maximum number of 3.7 × 10^6^ LL-37 molecules can cover the surface of a cell cultured in rich medium and a number of 1.8 × 10^6^ LL-37 molecules covers the surface of a cell cultured in minimal medium. The volume was calculated similarly and for cells growing in rich media a maximum number of 4.5 × 10^8^ LL-37 molecules remain at disposition to perturb the cell and possibly fill its cytoplasm. For cells cultured in minimal medium a number of 1.9 × 10^8^ LL-37 molecules is obtained. Spores can be bound by a maximum of 6.9 × 10^5^ and potentially “filled” by 1.7 × 10^8^ LL-37 molecules.

About 3.7 × 10^6^ LL-37 molecules are available to cover the cell wall surface and 3.7 × 10^6^ to cover the cell membrane surface leaving about 5.3 × 10^7^ LL-37 molecules still available to partition into the cytoplasm of a vegetative cell cultured in rich medium. The *B. subtilis* vegetative cell is obviously, not an empty object. The cytoplasm of the cell consist of DNA, RNA, ribosomes, proteins, a huge number of metabolites, and mobile genetic elements that reduces the available space within the cell. Cationic AMPs can potentially interact with all anionic macromolecules through electrostatic interaction (Table 3). Therefore, 5.3 × 10^7^ LL-37 molecules could be enough to target at least some of these components perturbing their normal function in cellular homeostasis.

**Table 3:** **Bionumbers of the ***Bacillus subtilis*** vegetative cell and spore composition**.

	***Bacillus subtilis***		**References**
	**Vegetative cell**	**Spore**	
**CELL WALL**			
Thickness	33.8 nm^#^	NA	
Teichoic acids	54%	SVC	Graham and Beveridge, 1994; Merchante et al., 1995
Peptidoglycan	46%	SVC	
			
**CELL MEMBRANE/INNER MEMBRANE OF SPORE**			
Protein	62%	SVC	Bishop et al., 1967
RNA	22%	SVC	
Phospholipids	16%	SVC	
Cardiolipin	10% of phospholipid	SVC	López et al., 2006
Phosphatidylglycerol	25% of phospholipid	SVC	
Phosphatidylethanolamine	50% of phospholipid	SVC	
Lysyl-phosphatidylglycerol	15% of phospholipid	SVC	
**CYTOPLASM**			
DNA	4215 kb	SVC	Logan and De Vos, 2015
RNA	NA	SVC	
Ribosomes	NA	SVC	
Protein	NA	SVC	
Mobile genetic elements	NA	SVC	
**SPORE COAT**			
Outer coat layer		40–90 nm	Henriques and Moran, 2000
Inner coat layer		20–30 nm	
Protein		10%	Munoz et al., 1978
Outer membrane		NA	
**CORTEX**			
Peptidoglycan		NA	

# *Values obtained from http://bionumbers.hms.harvard.edu/default.aspx*.

*Similar as vegetative cells (SVC) (Popham, 2002; Setlow, 2006)*.

*Not available (NA)*.

The estimation of a maximum of 3.7 × 10^6^ LL-37 molecules that can bind to the cell wall might also be an underestimation, since the cell wall is about 33.8 nm thick consisting of multiple peptidoglycan layers with imbedded teichoic acids. The muramyl peptides of peptidoglycan layers and the teichoic acids are anionic, therefore cationic AMPs will interact with all of these components in each layer before reaching the membrane. However, as long as the actual affinity constraints are not known it is unclear what the position is of the equilibrium reactions that the AMPs have with the various components of the cell. The actual situation maybe anything from a unidirectional reaction to one where the molecules diffuse more freely, attaching and detaching more or less stochastically to the binding sites.

The calculated values suggest that AMPs interact with more components of the cell than the cell membrane at the MIC value of LL-37 against *B. subtilis*. The primary target of the AMP is the membrane, but the phospholipids comprise only 16% of the total cell membrane (Refer to Table 2). The cell membrane also consist of 62% proteins, yet limited information is available about the interaction of AMPs with these components. It is likely that AMPs require the distortion of the membrane and the inactivation of macromolecules in conjunction to have a lethal effect. For instance, if a maximum of 3.7 × 10^6^ LL-37 molecules interact with a peptidoglycan layer of 1 nm, a total of 1.3 × 10^8^ LL-37 molecules could be bound to the 33.8 nm of cell wall leaving 6.5 × 10^7^ LL-37 molecules available to interact with the cell membrane. If the calculated maximum number of 3.7 × 10^6^ LL-37 molecules that can cover the cell membrane surface is deducted, 6.1 × 10^7^ LL-37 molecules are still available. The rapid association of the AMPs to the cell might be impairing the ability to observe all the possible targets of AMPs before cell death (Brogden, 2005).

AMPs are known to target germinated spores, i.e., when the cortex has been degraded, the DPA has been released and the core has been hydrated (Gut et al., 2008). The interaction that AMPs have with the proteins in the spore coat or with the outer membrane is not known, but AMPs do interact with the spore's inner membrane. The composition of the inner membrane and the core is similar to the cell membrane and cytosol of vegetative cells and it can be assumed that AMPs will interact similarly with spores as with vegetative cells (Popham, 2002; Setlow, 2006).

## Response of gram-positive bacteria to the presence of AMPs

Resistance of gram-positives against AMPs is infrequent and often information gathered about non-susceptible gram-positives have been used to understand their response to AMPs. Gram-positives respond to the presence of AMPs mainly through phenotypic alterations, which involves thickening of the cell wall, modification of the phospholipid composition, changing of the net surface charge, increasing the membrane fluidity, releasing proteinases to degrade the peptides and discharging amino acids into the environment to reduce hypo-osmotic stress.

In response to AMPs *S. aureus* and *Enterococcus faecalis* thicken their cell wall (Cui et al., 2003, 2006; Kramer et al., 2004; Yang et al., 2010; Arias et al., 2011; Bayer et al., 2014). The outer peptidoglycan layer of the thickened cell wall had a reduced cross-linked structure, which is thought to act like a molecular sieve preventing AMPs membrane passage (Cui et al., 2000). *S. aureus* also increased the content of non-amidated muropeptides in the cell wall peptidoglycan layer which increase the affinity of the cell wall for AMPs (Cui et al., 2000) thereby reducing the peptide's antimicrobial activity. However, a thickened cell wall is not always present in non-susceptible *S. aureus* strains (Yang et al., 2010) and can be temporary. *S. aureus* had a thickened cell wall in response to vancomycin, but the thickness reduced when vancomycin was removed from the culturing medium (Cui et al., 2003). The cell walls thickened again in this strain when vancomycin was reintroduced into the medium.

*S. aureus* can also alter its membrane fluidity in response to AMPs (Jones et al., 2008; Mishra et al., 2012). *S. aureus* strains decreased the carotenoid content in their cell membrane to reduce the membrane's fluidity in response to daptomycin (Mishra and Bayer, 2013). *Enterococcus faecium* had an increase of unsaturated fatty acids in its cell membrane in response to daptomycin which reduces membrane fluidity (Mishra et al., 2012). An increase of cyclopropane fatty acids in *E. faecium* was also observed, which is known to be involved in stabilizing the cell membrane (Glickman et al., 2000; Mishra et al., 2012).

Gram-positives alter their net surface charge by D-alanylation of the teichoic acids. This has been reported for *Streptococcus gordonii* (Chan et al., 2007), *C. difficile* (McBride and Sonenshein, 2011), *S. aureus* (Li et al., 2007a; Rose et al., 2012), and *B. subtilis* (Pietiäinen et al., 2005). D-alanylation which reduces the anionic charges of the teichoic acids, is regulated by the *dlt* operon (Peschel et al., 1999). The increased susceptibility to cationic AMPs after deletion of the *dlt* operon indicates that D-alanylation of the teichoic acids is one of the mechanisms of *S. aureus* to resists peptides. Similar results were obtained for *Streptococcus pneumoniae* (Kovacs et al., 2006), *B. cereus* (Abi Khattar et al., 2009), and *C. difficile* (McBride and Sonenshein, 2011). For more information about the D-alanylation process of teichoic acids refer to Neuhaus and Baddiley (2003).

**Figure 3:**
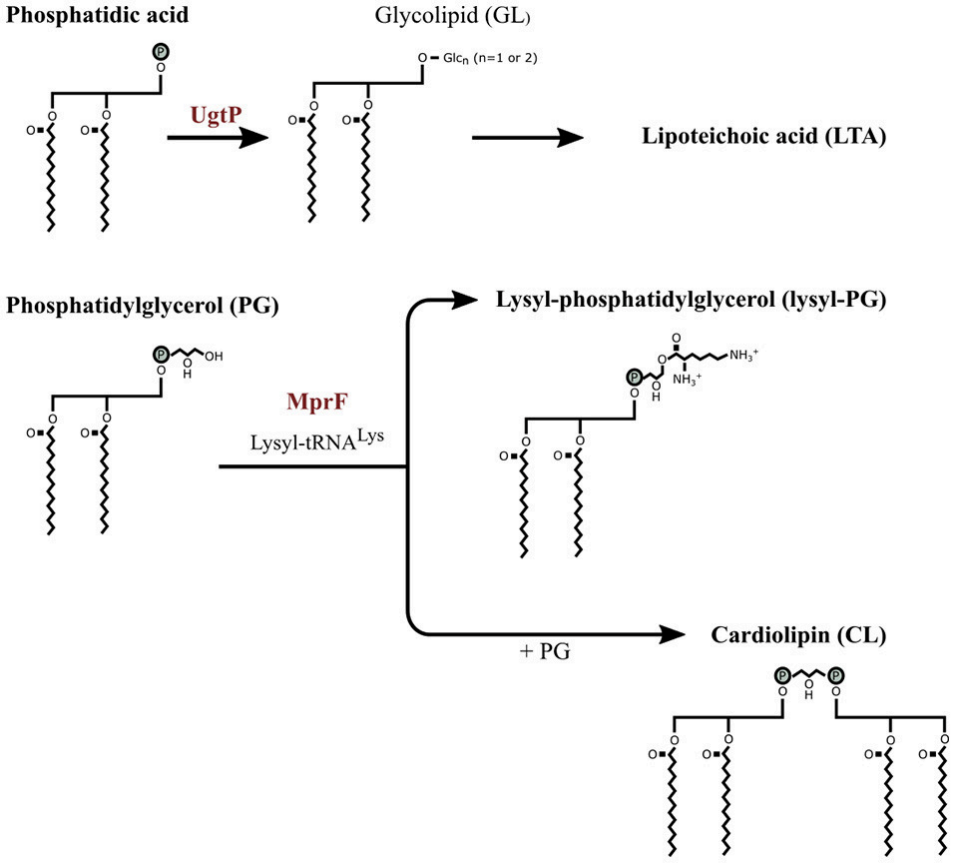
**The partial membrane lipid synthesis pathway of ***Bacillus subtilis*****. The precursor phosphatidic acid (PA), a common precursor for the membrane phospholipids, is dephosphorylated to diacylglycerol which is modified by UgtP through transferring one or two glucose molecules from UDP-glucose resulting in glycolipid (GL). Phosphotidylglycerol (PG) is converted to lysyl-PG when a lysyl group from lysyl-tRNA^Lys^ is transferred to PG by MprF. PG is also converted to cardiolipin by combining two PG molecules. Image adjusted from Salzberg and Helmann (2008).

The reduction in net surface charge can also be achieved by modifying the phospholipid composition of the membrane. *E. faecium* increased the lysyl-, alanyl- and arginyl-containing phospholipids and reduced its phosphatidylglycerol (PG) resulting in a lower negative cell membrane surface charge in response to daptomycin (Mishra et al., 2012). Similar results were obtained for *S. aureus* treated with daptomycin (Peleg et al., 2012) and *E. faecium* treated with mundticin KS, a bacteriocin (Sakayori et al., 2003). Salzberg and Helmann (2008) caused alterations of the membrane composition of *B. subtilis* by deleting various genes involved in the assembly of the membrane. The mutants lacking MprF were sensitive to nisin compared to the wild type and a deletion mutant lacking UgtP was sensitive to sublancin. MprF tranferes a lysyl group to PG from lysyl-tRNA^Lys^ to form lysyl-PG and cardiolipin synthase (CLS) condense two PG molecules to form CL (Figure 3) (Peschel and Sahl, 2006; Salzberg and Helmann, 2008). Deleting MprF prevents the synthesis of lysyl-PG and PG might be consequentially condensed to form CL or remain PG thus increasing the concentration of CL or PG in the membrane. In addition to reducing the net negative charge of the membrane, aminoacylated phosphatidylglycerols (e.g., lysyl-PG) stabilized the cell membrane (Cox et al., 2014). UgtP is involved in glycolipid synthesis and glycolipid is a precursor for LTA, but the cause of the increase in subtilin sensitivity of the deletion mutant lacking UgtP was unclear.

Previously, an mprF deletion mutant of *S. aureus* showed sensitivity to defensin and protegrin (Peschel et al., 2001). In *S. aureus* stains an increase in L-PG synthesis was only achieved after a point mutation in a certain regions in the *mprF* operon that caused an MprF gain-of-function phenotype (Yang et al., 2010; Bayer et al., 2014). The *S. aureus* stains with the MprF gain-of-function phenotype were also less sensitive to tPMPs and human neutrophil peptide 1 (hNP-1; defensin) from neutrophils (Bayer et al., 2014). MprF is present in various bacterial genomes and the modification is considered to be a general strategy against AMPs (Peschel and Sahl, 2006). However, reducing the net negative charge of the membrane has a limit and bacteria with reduced peptide susceptibility can still be killed by increasing the concentration of the AMPs.

The production of proteinases that degrade AMPs has been reported for certain bacterial species such as *Pseudomonas aeruginosa, E. faecalis, Proteus mirabilis*, and *Streptococcus pyogenes* (Schmidtchen et al., 2002). *S. aureus* produces a metalloproteinase (aureolysin) that degrades LL-37 rendering it inactive (Sieprawska-Lupa et al., 2004). *S. aureus* inhibits α-defensin by releasing staphylokinase that binds to the peptide to form a complex (Jin et al., 2004). *S. pyogenes* produce a cysteine proteinase called SpeB, that is bound to the cell surface and degrades LL-37 which made contact with the cell envelope (Nyberg et al., 2004). *P. aeruginosa, E. faecalis*, and *S. pyogenes* inactivated α-defensin by generating dermatan sulfate that binds to the peptide (Schmidtchen et al., 2001). These bacteria produce dermatan sulfate by releasing proteinases degrading dermatan sulfate-containing proteoglycans, such as decorin (Schmidtchen et al., 2001).

### *Bacillus subtilis* cell envelope stress response to AMPs

Phenotypic alterations brought about in response to AMPs are mainly due to the reaction of a signal-transducing regulatory system that induces countermeasures to repair damage and protect the cell in response to cell envelope alterations and abnormalities (Jordan et al., 2008). *B. subtilis* regulates its stress response with the extracytoplasmic function (ECF) sigma factors and a two component system (TCS) (Jordan et al., 2008; Kingston et al., 2011). Both are signaling systems that consist of a membrane-bound sensor kinase and a response regulator (Jordan et al., 2008). The regulator remains inactive in conditions that do not cause cell envelope stress, but as soon as envelope stress is detected the regulator is activated and induces the expression of its target genes.

In response to sublethal concentrations of LL-37, *B. subtilis* activated the SigM and SigW regulons controlled by the ECF sigma factors (Pietiäinen et al., 2005). The SigM regulon is activated by cell wall antibiotics, acidic pH, heat, ethanol, superoxide, and cell envelope stress (Cao and Helmann, 2002; Minnig et al., 2003; Thackray and Moir, 2003). It is involved in activating various genes involved in cell wall biosynthesis, cell division and cell shape, DNA damage response and detoxification enzymes (Eiamphungporn and Helmann, 2008). SigW is also induced by the presence of cell wall active antibiotics, such vancomycin, and by the membrane active AMP PG-1 and alkaline shock (Wiegert et al., 2001; Cao and Helmann, 2002; Pietiäinen et al., 2005). SigW regulates the alteration of the fatty acid composition of the cell membrane that results in a reduced or increased membrane fluidity (Kingston et al., 2011). Peptide PG-1 activated SigM and SigX in *B. subtilis* (Pietiäinen et al., 2005). SigX is involved in regulating the overall net charge of the envelope as it steers the *dltABCDE* and *pssA-ybfM-psd* operons (Cao and Helmann, 2004). As mentioned before, the *dlt* genes control D-alanylation of the LTA and PssA/Psd catalyzes the synthesis of phosphatidylethanolamine (PE). The combined effect is a less negatively charged cell membrane (Cao and Helmann, 2004).

In addition to the activation of ECF sigma factors, two component systems were activated. LL-37 activated the two component system YxdJK TCS and LiaRS (YvqCE) TCS (Pietiäinen et al., 2005). The two-component system activated by *B. subtilis* is dependent on the peptide. For instance, the membrane targeting peptide PG-1 activated only LiaRS (YvqCE) TCS and not the YxdJK TCS (Pietiäinen et al., 2005). The LiaRC TCS and ECF sigma factors are a cell wall antibiotic-response system responsible for preserving cell envelope integrity and prevention of cell envelope damage (Jordan et al., 2008). These systems' regulon is diverse and does not mediate a specific antibiotic resistance (Jordan et al., 2008). The role that these systems have are to maintain homeostasis (Jordan et al., 2008).

*B. subtilis* has three TCS/ABC transporter modules; the BceRS-BceAB, the YvcPQ-yvcRS, and YxdJK-yxdLM systems (Joseph et al., 2002; Mascher, 2006). The ABC transporter involved in the BceRS system is specific for the removal of bacitracin from the cell envelope (Mascher et al., 2003; Ohki et al., 2003). The process of bacitracin removal is still unknown, but a hydrophobic vacuum cleaner model has been suggested which involves the removal of bacitracin by a transporter directly from the phospholipid bilayer to extracellular environment (Bolhuis et al., 1996; Ohki et al., 2003). BceRS was also induced by other cell wall synthesis inhibiting peptides such as plectasin, mersacidin and actagardine (Staroń et al., 2011). The YxdJK-yxdLM system is activated by LL-37 (Pietiäinen et al., 2005). The role of this system has not been identified yet (Jordan et al., 2008). The YvcPQ-yvcRS system is activated in response to lipid II-binding lantibiotics such as nisin and gallidermin (Staroń et al., 2011), but its role is unknown.

Other genes that were also upregulated in response to LL-37 are the *bcrC* (*ywoA*) gene involved in cell wall synthesis and bacitracin resistance (Pietiäinen et al., 2005), *pbpE* (penicillin-binding protein) that is known to be induced by cell wall stress (Cao et al., 2002) and *dltB* which is like the *dlt* operon (Pietiäinen et al., 2005). Concluding, *B. subtilis* respond to the onslaught of AMPs by reducing the net negative charge of the cell surface, by attempting to maintain cell envelope homeostasis and by removing the AMPs from the cell surface.

### *Staphylococcus aureus* cell envelope stress response to AMPs

To obtain insight whether other gram-positive bacteria responds similarly to *B. subtilis* to the presence of AMPs, the response of *Staphylococcus* sps. was reviewed. In response to cationic peptides, *S. epidermis* and *S. aureus* showed to activate, similar to *B. subtilis*, a two-component sensor/regulator system called the *aps* system (Li et al., 2007a,b; Yang et al., 2012). The *aps* (*graRS*) regulated gene loci have been associated with resistance against cationic AMPs such as defensin, indolicin and LL-37. The *aps* system consist of three components known as ApsS, ApsR, and ApsX (Li et al., 2007a,b). The *aps* system regulates the *dlt* operon, the *mprF* gene, and *vraFG*, a putative ABC transporter-dependent efflux pump, thought to be exclusively involved in AMP resistance (Li et al., 2007a,b; Yang et al., 2012). In addition to *aps* system role as regulatory system, the extracellular sensing loop of ApsS has a high density of negative charges that binds to peptides, directly rendering the AMP inactive (Li et al., 2007a,b). Yang et al. (2012) reported that these phenotypic changes brought about by the *aps* system to a single AMP could also cause cross-resistance to another AMP that is structurally different.

## Conclusion

In conclusion, cationic AMPs can cause membrane perturbation, which can either cause physical damage to the membrane or may leave the membrane intact. In both cases the membrane potential can be lost or ion efflux can take place. Peptides that traverse the membrane do not always cause efflux of ions. Cationic AMPs have been shown to target the septum and new poles (Skerlavaj et al., 1999; Sochacki et al., 2011; Rangarajan et al., 2013), and to cause delocalization of membrane-bound proteins (Wenzel et al., 2014) or localization of proteins at incorrect sites at the membrane (Pogliano et al., 2012). The latter has been confirmed with the formation of abnormal septa (Friedrich et al., 2000; Nakajima et al., 2003; Pogliano et al., 2012). Cationic AMPs do bind to macromolecules (DNA, RNA or protein) and thus prevent the normal functioning of the cell (Falla et al., 1996; Miyazaki et al., 1999; Xiong et al., 2005; Dangel et al., 2013; Ghosh et al., 2014). AMPs also inhibited cell wall biosynthesis without causing membrane damage by binding to lipid II and induce autolysis. Additionally, antimicrobial activity of AMPs have been observed against spores but only if these have previously germinated.

The bionumbers and bioestimates was utilized to obtain a rough order of magnitude estimation of the number of AMPs that can target the vegetative *B. subtilis* cell or spore at the MIC values. The calculations suggested that a maximum of 6.0 × 10^7^ LL-37 molecules can target each vegetative cell or spore at the MIC value of 1 μM. When the theoretical surface area and volume of the vegetative cell or spore was calculated, a maximum of 3.7 × 10^6^ LL-37 molecules was estimated to cover the surface area of the vegetative cell in a hexagonal packing formation when the cell has been cultured in rich medium. When this maximum number of LL-37 molecules were deducted from the cell wall and cell membrane surface area, the results showed that at MIC more LL-37 molecules are available to target the vegetative cell than what is required for membrane binding. Even when the bionumber of the cell wall was also taken into consideration. These calculations suggest that LL-37 targets more cell components than the cell membrane, even if some of the peptides should get trapped in the thick cell wall. Hence it is highly likely that intracellular macromolecules are also targeted at the MIC value. However, the LL-37 molecules might also be bound to the surface of the test tube or the well of the microtiter plate, or be bound to components of the culturing medium. If this is the case, than the actual number of LL-37 molecules targeting the cell may obviously be less than what has been calculated.

The response of some gram-positives to the attack by cationic AMPs is to thicken their cell wall thus entrapping peptides or even to create an affinity trap. The bacteria may reduce their membrane fluidity to prevent insertion of peptides into the membrane or to stabilize it. Released amino acids may reduce the hypo-osmotic pressure caused by the interaction of the AMPs with the cell membrane. Some gram-positives reduce their anionic net charge by modifying their phospholipid composition or by D-alanylating their teichoic acids to repel the cationic peptides. Most of these physical alterations have been confirmed though transcriptomic or proteomic approaches and it appears that the bacteria respond to AMPs by activating their general cell envelope stress response and by removing the AMPs from the cell surface.

However, to increase our understanding of the mode of action of AMPs or the response of bacteria to AMPs more information is required. For now the use of fluorescence microscopy and real-time single cell live imaging has already given more information concerning the mode of action of AMPs than what TEM or SEM have initially provided (Sochacki et al., 2011; Pogliano et al., 2012; Barns and Weisshaar, 2013; Rangarajan et al., 2013). Furthermore, a new area of considerable importance for the medical field as well as for food safety is the action of AMPs on bacterial spores. The limited information available on the mode of action of AMPs against spores indicates AMPs target only germinated spores, but there may be AMPs with different modes of anti-spore activity. Quantitatively there is a need for data, for example pre-steady state kinetics, on the interaction strength or affinity constraints of AMPs with the various cell components. Also the further development of traceable AMPs that retain their normal antimicrobial activity will benefit our attempt to increase our understanding of the mode of action of AMPs.

A better understanding of the modes of action of various AMPs will improve the design of AMPs aimed for use as antimicrobial agents. Having a solid knowledge base of the response of different bacteria to these peptides will provide information on what to expect when bacteria do develop resistance during the clinical use of AMPs or their possible application as preservative in the food chain.

## Author contributions

SO as main contributor. SB and SZ contributed equally as editors of the manuscript.

### Conflict of interest statement

The authors declare that the research was conducted in the absence of any commercial or financial relationships that could be construed as a potential conflict of interest.
